# Bilateral Tubo-Ovarian Abscesses Associated with Enterococcal Translocation in Decompensated Cirrhosis: A Case Report

**DOI:** 10.3390/reports9020116

**Published:** 2026-04-10

**Authors:** Noor Albusta, Hussain Alrahma

**Affiliations:** 1 Department of Internal Medicine, Lahey Hospital and Medical Center, Burlington, MA 01805, USA; 2 Department of Gastroenterology and Hepatology, Salmaniya Medical Complex, Manama P.O. Box 12, Bahrain; hrahma1@hospitals.gov.bh

**Keywords:** cirrhosis, tubo-ovarian abscess, secondary bacterial peritonitis, cirrhosis associated immune dysfunction, ascites

## Abstract

**Background and Clinical Significance:** Cirrhosis-associated immune dysfunction (CAID) is characterized by impaired innate and adaptive immune responses, gut dysbiosis, and increased bacterial translocation, predisposing patients to severe and atypical infections. While spontaneous bacterial peritonitis and other intra-abdominal infections are well-recognized complications of cirrhosis, extraintestinal infectious manifestations related to bacterial translocation are less commonly described. A tubo-ovarian abscess (TOA) typically arises from ascending pelvic infections associated with pelvic inflammatory disease and is rarely reported in patients with cirrhosis without gynecologic risk factors. Thus, recognizing unusual infectious presentations in cirrhotic patients is important given their functionally immunocompromised state. **Case Presentation:** We report a 46-year-old woman with previously undiagnosed alcohol-related cirrhosis who presented with sepsis and abdominal pain. She had no prior gynecologic history or known risk factors for pelvic inflammatory disease. Contrast-enhanced computed tomography (CT) demonstrated bilateral tubo-ovarian abscesses. Image-guided percutaneous drainage was performed, and cultures from both ascitic fluid and bilateral adnexal collections grew *Enterococcus faecium*, supporting a shared intra-abdominal source of infection and suggesting transperitoneal dissemination via infected ascitic fluid as a plausible mechanism, although an ascending genital tract source cannot be fully excluded. The patient was treated with targeted intravenous antibiotics and drainage with subsequent clinical improvement. **Conclusions:** This case highlights bilateral tubo-ovarian abscesses as a rare infectious complication of cirrhosis-associated immune dysfunction. In cirrhotic patients presenting with sepsis and intra-abdominal pathology, clinicians should consider atypical infection pathways related to bacterial translocation among the differential mechanisms of spread. Thus, recognizing cirrhosis as a functionally immunocompromised state is essential for the timely diagnosis and management of unusual infections.

## 1. Introduction and Clinical Significance

Cirrhosis represents a state of acquired immune dysfunction, termed cirrhosis-associated immune dysfunction (CAID), which is characterized by impaired innate and adaptive immune responses, increased intestinal permeability, gut microbiome alterations, and enhanced bacterial translocation from the gastrointestinal tract [1,2,3]. These pathophysiologic changes predispose patients with cirrhosis to a broad spectrum of infections. While common complications such as spontaneous bacterial peritonitis (SBP), urinary tract infections, and pneumonia are well-recognized, cirrhotic patients are also vulnerable to severe, disseminated, and atypical infections involving uncommon anatomical sites due to their functionally immunocompromised state [2,3]. Secondary bacterial peritonitis differs from SBP in that it arises from an identifiable intra-abdominal source, often with polymicrobial infection or enteric organisms, such as *Enterococcus* species, and typically requires both antimicrobial therapy and source control [4,5]. In contrast, a tubo-ovarian abscess (TOA) most commonly develops as a complication of pelvic inflammatory disease caused by ascending genital tract infection, usually affecting younger women with identifiable sexually transmitted infection risk factors. A bilateral TOA in middle-aged women without these risk factors is uncommon and suggests alternative mechanisms of infection, including possible transperitoneal spread or hematogenous seeding [5,6].

Thus, recognizing atypical infection pathways in patients with cirrhosis is clinically important because delayed diagnosis and inadequate source control may lead to rapid clinical deterioration in this high-risk population. We report a rare case of bilateral tubo-ovarian abscesses occurring in a patient with decompensated alcohol-related cirrhosis without traditional gynecologic risk factors. This case highlights cirrhosis-associated immune dysfunction as a potential predisposing factor for unusual intra-abdominal infections and underscores the importance of early recognition and timely source control in patients with advanced liver disease.

## 2. Case Presentation

A 46-year-old woman with no known prior medical history presented to the emergency department with progressive abdominal distension, lethargy, and jaundice. She had not engaged with medical care and had not seen a physician in over a decade prior to this presentation. Her medical history indicated chronic alcohol use with daily consumption of approximately one bottle of wine for 4–5 years, with cessation three months prior to presentation. She denied any history of viral hepatitis, autoimmune diseases, metabolic disorders, or a family history of liver disease. The patient had no known history of pelvic inflammatory disease, sexually transmitted infections, or recent gynecologic procedures. Upon initial evaluation, her vital signs were as follows: a body temperature of 38.9 °C, a heart rate of 118 beats per minute, a blood pressure of 92/58 mmHg, a respiratory rate of 24 breaths per minute, and 94% oxygen saturation on room air. A physical examination demonstrated marked abdominal distension with diffuse tenderness, shifting dullness consistent with ascites, scleral icterus, and stigmata of chronic liver disease including palmar erythema and spider angiomata, while a pelvic examination revealed no vaginal discharge or cervical motion tenderness. Additionally, laboratory evaluation revealed leukocytosis (24.8 × 10^9^/L), thrombocytopenia (62 × 10^9^/L), acute kidney injury (creatinine 1.8 mg/dL), hyperbilirubinemia (9.4 mg/dL), coagulopathy (INR 2.1), and elevated lactate levels (4.6 mmol/L) (Table 1). Her serum sodium concentration was 136 mmol/L, and the calculated MELD-Na score was 29.

She was subsequently admitted for the evaluation and management of sepsis and newly diagnosed decompensated cirrhosis, likely secondary to alcohol use disorder, given her extensive chronic alcohol consumption. Diagnostic paracentesis demonstrated cloudy ascitic fluid with an absolute neutrophil count of 6700 cells/µL and a serum-ascites albumin gradient of 1.6 g/dL. *Enterococcus faecium* grew from her ascitic fluid culture, raising concern for secondary bacterial peritonitis given the neutrophil count and isolation of an enteric organism (Table 2).

**Figure 1:**
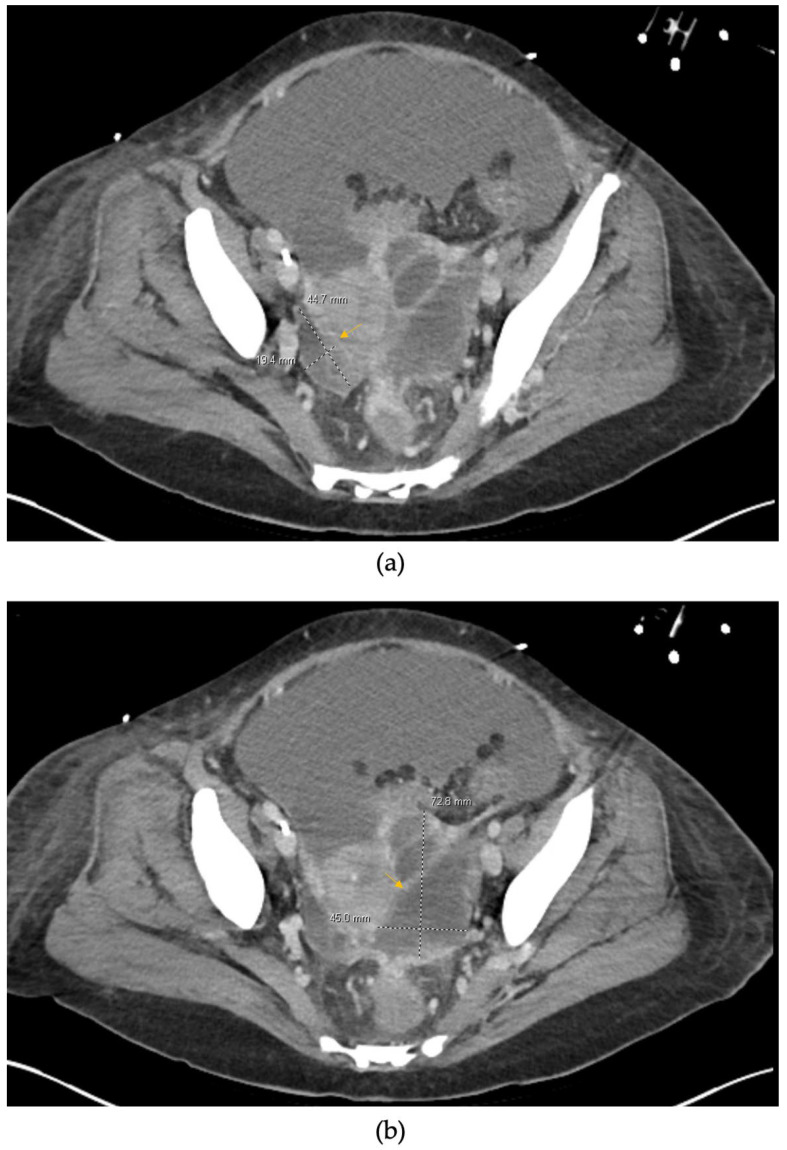
Axial contrast-enhanced computed tomography (CT) images of the pelvis demonstrating bilateral adnexal multiloculated fluid collections consistent with tubo-ovarian abscesses in the presence of large-volume ascites. Yellow arrows indicate the adnexal collections. (**a**) Right adnexal multiloculated collection measuring approximately 4.5 × 1.9 cm with surrounding inflammatory changes. (**b**) Larger left adnexal multiloculated collection measuring approximately 7.3 × 4.5 cm, demonstrating complex fluid attenuation and adjacent inflammatory stranding.

Contrast-enhanced computed tomography (CT) of the abdomen and pelvis revealed a nodular cirrhotic liver, portal hypertension stigmata, large-volume ascites, and bilateral adnexal multiloculated collections (left: 7.3 × 4.5 cm; right: 4.5 × 1.9 cm) with surrounding inflammatory changes (Figure 1).

**Figure 2:**
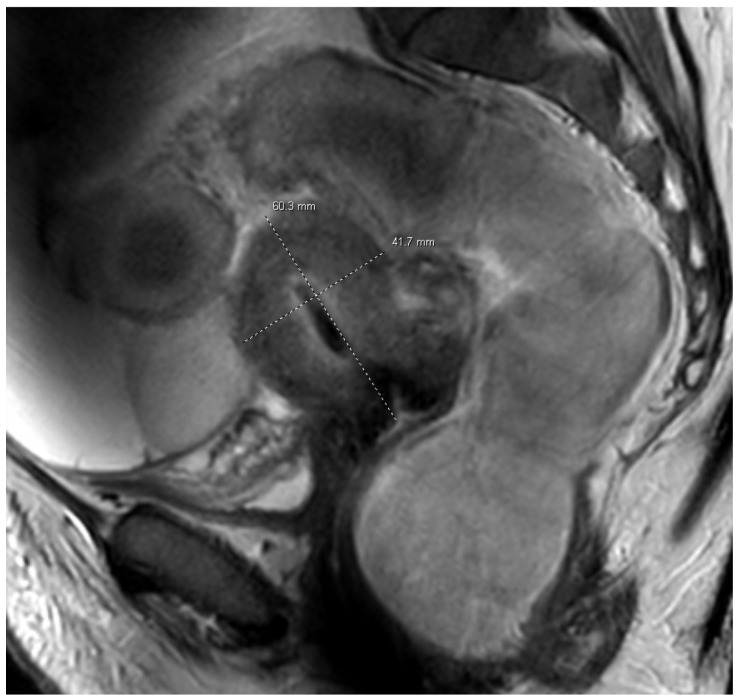
Sagittal pelvic magnetic resonance imaging demonstrating a thick-walled tubular left adnexal collection measuring approximately 6.0 × 4.2 cm, with imaging features consistent with a tubo-ovarian abscess or pyosalpinx, supporting an infectious etiology rather than malignancy. Restricted diffusion and thick peripheral enhancement favored abscess formation.

Given the presence of adnexal masses in the setting of ascites, the differential diagnosis included tubo-ovarian abscesses, pyosalpinx, hydrosalpinx, and ovarian malignancy. Serum CA-125 was elevated at 109 U/mL, further raising concern for a possible ovarian malignancy in the context of adnexal masses and ascites. The patient denied pelvic pain, vaginal discharge, and nucleic acid amplification testing for Neisseria gonorrhoeae, and her Chlamydia trachomatis result was negative. For further characterization and to better delineate infectious versus malignant etiologies, pelvic magnetic resonance imaging was obtained. Pelvic MRI demonstrated a thick-walled, multiloculated collection with restricted diffusion in the left adnexa consistent with TOAs, without features suggesting malignancy (Figure 2).

Following multidisciplinary discussion, the interventional radiologist performed bilateral percutaneous drainage for source control. Abscess cultures grew *Enterococcus faecium* with an antimicrobial susceptibility profile concordant with the ascitic fluid isolate, supporting a shared source of infection.

The patient was initially treated empirically with intravenous piperacillin–tazobactam and vancomycin for suspected secondary bacterial peritonitis. The cultures from both ascitic fluid and bilateral adnexal abscesses grew *Enterococcus faecium* that was ampicillin-resistant but vancomycin-susceptible, and antimicrobial therapy was continued with intravenous vancomycin. The isolate’s antimicrobial susceptibility pattern is presented in Table 3.

She completed 14 days of therapy following the CDC’s guidelines for managing tubo-ovarian abscesses [7]. The patient demonstrated progressive clinical improvement with fever resolution, declining leukocytosis, and lactate normalization, and she was discharged under a stable condition with outpatient follow-up.

## 3. Discussion

This case most likely represents a shared intra-abdominal infectious process involving both infected ascitic fluid and bilateral tubo-ovarian abscesses, rather than proving a single definitive direction of spread. To our knowledge, this is an extremely rare presentation of bilateral TOAs in a patient with decompensated cirrhosis without traditional gynecologic risk factors. Cirrhosis impairs neutrophil chemotaxis, phagocytosis, complement activity, and cytokine signaling, while portal hypertension and gut dysbiosis increase intestinal permeability and bacterial translocation [1]. These changes predispose patients to severe, disseminated, and atypical infections [1,2,3].

The isolation of *Enterococcus faecium* from both ascitic fluid and bilateral adnexal abscesses supports the possibility of transperitoneal dissemination as the mechanism of infection. However, alternative mechanisms should also be considered. Tubo-ovarian abscesses most commonly result from ascending genital tract infection associated with pelvic inflammatory disease [6]. However, the absence of pelvic symptoms, a lack of sexually transmitted infection risk factors, and bilateral involvement make this mechanism less likely in the present case. Hematogenous dissemination is another potential pathway, although the concordant isolation of *Enterococcus faecium* from ascitic fluid and abscess cultures favors a shared peritoneal source.

The distinction between SBP and secondary bacterial peritonitis is clinically important because management differs substantially. SBP is typically a monomicrobial infection of ascitic fluid without a surgically treatable source, whereas secondary bacterial peritonitis arises from an identifiable intra-abdominal focus and generally requires source control in addition to antibiotics [4,5]. In this patient, several features favored secondary bacterial peritonitis over uncomplicated SBP: a markedly elevated ascitic neutrophil count, culture growth of an enteric organism, severe systemic toxicity at presentation, and subsequent imaging evidence of bilateral drainable pelvic abscesses. Although classic descriptions of secondary peritonitis often emphasize polymicrobial cultures, monomicrobial infection does not exclude the diagnosis when a clear anatomic source is present.

In cirrhosis, bacterial translocation occurs through gut–vascular barrier dysfunction, allowing bacteria to enter the peritoneal cavity where ascites serves as a conduit for dissemination to adjacent structures including the adnexa [1,2,3]. Bilateral TOAs in middle-aged women without sexually transmitted infection risk factors are uncommon and have been primarily described in immunocompromised patients [6,8].

The gut microbiome in cirrhosis demonstrates characteristic enrichment of Enterococcus species with depletion of beneficial autochthonous bacteria—changes that worsen with disease progression [3]. Alterations include a loss of genetic diversity, a decrease in autochthonous species, and enrichment with pathobionts including *Enterococcaceae* [1,3]. This altered microbial landscape predisposes patients to infections not traditionally associated with SBP or gynecologic infections.

Published literature also supports that TOA is usually a complication of PID in reproductive-age women, but atypical cases do occur in patients without standard sexual risk factors or with alternative intra-abdominal sources [9,10]. This broader context supports interpreting the present case as an uncommon but biologically plausible manifestation of infection in advanced cirrhosis.

Elevated CA-125 posed a diagnostic challenge given the presence of adnexal masses and ascites. CA-125 is produced by mesothelial cells, and elevated levels in patients with cirrhosis reflect peritoneal inflammation rather than malignancy. Moreover, studies demonstrate that 94–98% of cirrhotic patients with ascites have elevated CA-125 levels, correlating with ascitic volume and declining after paracentesis [11]. Thus, raising awareness of this phenomenon helps prevent unnecessary oncologic workup.

Standard TOA management includes antimicrobial therapy with early consideration of drainage. According to CDC guidelines, 14 days of antimicrobial therapy is recommended for TOAs, with parenteral therapy continued until clinical improvement followed by a transition to oral therapy [7]. Image-guided drainage combined with antibiotics is now considered first-line therapy for TOAs, particularly in patients with large, bilateral, or complex abscesses. Recent systematic reviews demonstrate that image-guided drainage achieves success rates of 90–100%, with lower complication rates and shorter hospital stays compared to laparoscopic drainage or antibiotics alone [6,12]. In advanced cirrhosis with portal hypertension, coagulopathy, and hemodynamic instability, even minimally invasive procedures carry substantial risks. However, prompt image-guided percutaneous drainage can provide definitive source control while avoiding the risks of open surgical intervention [13]. In this case, multidisciplinary planning enabled safe drainage despite advanced cirrhosis, resulting in a favorable clinical response.

## 4. Conclusions

This case expands the recognized spectrum of CAID-associated infections to include bilateral tubo-ovarian abscesses occurring concurrently with infected ascitic fluid containing Enterococcus faecium. Although the exact route of spread cannot be established definitively from a single case, transperitoneal dissemination related to bacterial translocation represents a plausible explanation in the setting of decompensated cirrhosis and large-volume ascites. Clinicians should consider secondary sources when ascitic fluid characteristics are atypical for SBP and pursue timely source control when feasible.

## Figures and Tables

**Table 1:** Laboratory findings on admission.

Laboratory Parameter	Value	Reference Range
White blood cell count	24.8 × 10^9^/L	4.0–11.0 × 10^9^/L
Neutrophils (absolute)	20.1 × 10^9^/L	2.0–7.5 × 10^9^/L
Hemoglobin	9.2 g/dL	12.0–16.0 g/dL
Platelet count	62 × 10^9^/L	150–400 × 10^9^/L
Sodium	136 mmol/L	135–145 mmol/L
Creatinine	1.8 mg/dL	0.6–1.2 mg/dL
AST (aspartate aminotransferase)	156 U/L	10–40 U/L
ALT (alanine aminotransferase)	68 U/L	7–56 U/L
Total bilirubin	9.4 mg/dL	0.2–1.2 mg/dL
Albumin	2.1 g/dL	3.5–5.0 g/dL
Alkaline phosphatase	142 U/L	44–147 U/L
INR	2.1	0.8–1.2
Lactate	4.6 mmol/L	0.5–2.0 mmol/L

Abbreviations: AST, aspartate aminotransferase; ALT, alanine aminotransferase; INR, international normalized ratio.

**Table 2:** Ascitic fluid analysis.

Ascitic Fluid Parameter	Value	Reference Range/Interpretation
Appearance	Cloudy	Clear to pale yellow (normal)
Absolute neutrophil count	6700 cells/µL	<250 cells/µL (normal)
Total protein	1.2 g/dL	Low (<1.5 g/dL in cirrhosis)
Albumin (ascitic)	0.5 g/dL	Used for SAAG calculation
SAAG	1.6 g/dL	≥1.1 → portal hypertension
Culture	*Enterococcus faecium*	Sterile (normal)

Abbreviations: SAAG, serum-ascites albumin gradient.

**Table 3:** Antibiotic susceptibility profile of *Enterococcus faecium* isolated from ascitic fluid and tubo-ovarian abscesses.

Antibiotic	Susceptibility	MIC (µg/mL)
Ampicillin	Resistant	>32
Vancomycin	Susceptible	1
Linezolid	Susceptible	2
Daptomycin	Susceptible	2
Ciprofloxacin	Resistant	>4

Abbreviations: MIC, minimum inhibitory concentration.

## Data Availability

The original contributions presented in this study are included in the article. Further inquiries can be directed to the corresponding author.

## Abbreviations

The following abbreviations are used in this manuscript:

CAID	Cirrhosis-Associated Immune Dysfunction
TOA	Tubo-Ovarian Abscess
SBP	Spontaneous Bacterial Peritonitis
CT	Computed Tomography
MRI	Magnetic Resonance Imaging
MELD-Na	Model for End-Stage Liver Disease—Sodium Score
CA-125	Cancer Antigen 125
CDC	Centers for Disease Control and Prevention
IR	Interventional Radiology
NAAT	Nucleic Acid Amplification Test
SAAG	Serum-Ascites Albumin Gradient
MIC	Minimum Inhibitory Concentration
